# Evaluation and Comparison of Respiratory Muscular Strength, Functionality, and Pelvic Floor in the Immediate Postpartum of Normal and Cesarean Birth

**DOI:** 10.1055/s-0043-1768457

**Published:** 2023-04-27

**Authors:** Carolina Martins da Rosa, Thariny Huesken Dockhorn, Juliana Rezende Cardoso, Soraia Genebra Ibrahim Forgiarini, Luiz Alberto Forgiarini Junior, André Luiz Lisboa Cordeiro

**Affiliations:** 1 Centro Universitário Metodista, Porto Alegre, RS, Brazil; 2 Universidade Federal de Ciências da Saúde, Porto Alegre, RS, Brazil; 3 Universidade La Salle, Botucatu, SP, Brazil; 4 Centro Universitário Nobre, Feira de Santana, BA, Brazil

**Keywords:** Postpartum period, Cesarean section, Pelvic floor, Physical performance, Período pós-parto, Cesárea, Assoalho pélvico, Desempenho físico

## Abstract

**Objective:** To evaluate and compare peripheral, pelvic floor, respiratory muscle strength, and functionality in the immediate puerperium of normal delivery and cesarean section.

**Methods:** This is a cross-sectional study that verified respiratory, pelvic floor, peripheral, and functional muscle strength through manovacuometry, pelvic floor functional assessment (PFF), dynamometry, and the Time Up and Go (TUG) test, respectively. The groups were divided according to the type of delivery, into a cesarean section group and a normal parturition group.

**Results:** The sample was composed of 72 postpartum puerperae, 36 of normal parturition, and 36 of cesarean section, evaluated before hospital discharge, mean age ranged from 25.56 ± 6.28 and 28.57 ± 6.47 years in puerperae of normal parturition and cesarean section respectively. Cesarean showed higher pelvic floor strength (PFF) compared to normal parturition (p < 0.002), but puerperae from normal delivery showed better functionality (p < 0.001). As for peripheral muscle strength and respiratory muscle strength, there was no significance when comparing the types of parturirion.

**Conclusion:** There is a reduction in pelvic muscle strength in puerperae of normal delivery and a decrease in functionality in puerperae of cesarean section.

## Resumo

**Objetivo:** Avaliar e comparar o pavimento pélvico periférico, a força muscular respiratória e a funcionalidade no puerpério imediato do parto normal e da cesariana.

**Métodos:** Este é um estudo transversal que verificou a força muscular respiratória, pavimento pélvico, periférico e funcional através da manovacuometria, avaliação funcional do pavimento pélvico (PFF), dinamometria, e o teste Time Up e Go (TUG), respectivamente. Os grupos foram divididos de acordo com o tipo de parto, num grupo de cesariana e num grupo de parto normal.

**Resultados:** A amostra foi composta por 72 puérperas pós-parto, 36 de parto normal e 36 de cesariana, avaliados antes da alta hospitalar, a idade média variou entre 25,56 ± 6,28 e 28,57 ± 6,47 anos em puérperas de parto normal e cesariana, respectivamente. A cesariana mostrou maior resistência do pavimento pélvico (TFP) em comparação com o parto normal (p < 0,002), mas as puérperas de parto normal mostraram melhor funcionalidade (p < 0,001). Quanto à força muscular periférica e à força muscular respiratória, não houve significado ao comparar os tipos de parto.

**Conclusão:** Há uma redução da força muscular pélvica em puérperas de parto normal e uma diminuição da funcionalidade em puérperas de cesarianas.

## Introduction

The puerperium or postpartum is defined by a variable temporal period in which the changes that occur due to pregnancy and childbirth in the woman's body return to their pre-pregnancy state. 1 During pregnancy and the puerperium several physiological adaptations and changes occur in the cardiovascular, endocrine, tegumentary, urinary, gastrointestinal, respiratory, and musculoskeletal systems in the maternal organism, which consequently triggers anatomical and functional changes. 2 3

Normally the return to pre-pregnancy conditions, such as regression and recovery of the uterine musculature and vaginal mucosa, occurs in an average of six to eight weeks. 1 Thus, pregnancy and the route of delivery are risk factors for alterations in the pelvic floor (PF) muscle strength and may also lead to some changes in anatomical position and pelvic muscles, as well as changes in the viscera and perineum. 2 4 Besides the mechanical and biochemical alterations, there are also important adaptations and modifications in lung volumes and capacities, due to the growth of the uterus, causing modifications in the resting position of the diaphragm and in the configuration of the chest wall, thus interfering in the strength of the inspiratory and expiratory respiratory muscles. 5 6 7 8

The type of parturition is linked to the woman's recovery time and, therefore, may contribute to functional limitations. The change of the female body and the return of body systems to pre-pregnancy may impair her activities of daily living (ADLs). 9 Both parturitions can interfere in the functionality of the puerpera, in vaginal childbirth, part of the woman may suffer some type of perineal trauma due to spontaneous lacerations and/or episiotomies, these lacerations may be associated with morbidities that can interfere with the performance of usual activities. 10 11 As for the cesarean section, the abdominal incision causes trauma to the woman's body, generating pain in the scar, less bowel movement, accumulation of gases, and restricted physical mobility, which can cause losses in postpartum recovery. 12

Thus, in view of the above-mentioned alterations, the objective of this study was to evaluate and compare respiratory muscle strength, pelvic floor muscle strength, peripheral muscle strength, and functionality in women who underwent normal parturition and cesarean section.

## Methods

This is a cross-sectional study, developed at Hospital Fêmina, belonging to Grupo Hospitalar Conceição, in Porto Alegre/RS, carried out from October 2016 to May 2017. The project was approved by the Ethics and Research Committee of the Centro Universitário Metodista - IPA with opinion number 1.709.047.

Inclusion criteria were women over 18 years of age, primiparous or multiparous, not implying the number of previous pregnancies, up to the 4th day of immediate puerperium from cesarean or normal delivery, without previous respiratory diseases, with a minimum gestational age of 37 weeks and hemodynamically stable. The exclusion criteria were inability to perform any of the tests, previous history of abortions or surgeries, twin pregnancy, and diagnosis of uncontrolled hypertension or diabetes mellitus. All patients included in the study signed an informed consent form.

For the data collection procedure, the medical records were evaluated as a preselection, and demographic data were collected. The assessments were performed at a single time, following the order of respiratory muscle strength assessment, pelvic floor strength assessment, peripheral strength assessment, and lastly, functionality assessment. Vital signs, as well as weight and height, were collected from the patient's hospital chart. The postpartum women were intentionally allocated according to the type of parturition.

Respiratory muscle strength was assessed by manovacuometry, with an analog manovacuometer (MVD 120, Globalmed, Porto Alegre, RS). To evaluate Maximum Inspiratory Pressure (MIP), the pregnant woman was asked to exhale up to residual volume and then, immediately, to place the mouthpiece, inhaling up to total lung capacity, maintaining two seconds of sustained force, and finally removing the mouthpiece. And, to evaluate the Maximum Expiratory Pressure (MEP), the participant performed the reverse procedure, requesting at the end, an expiration up to the residual volume. During the measurements, the pregnant women were properly positioned in a sitting position, there was a rest interval between one evaluation and the other, a nose clip was also used in both maneuvers to better determine the pulmonary pressures, and each measurement was requested three repetitions, and the best result was used. With the results obtained, the predictive equations of maximum respiratory pressures proposed by another study were performed. 13

Pelvic floor muscle strength was measured through the pelvic floor assessment (FPA), which was performed with the patient in a gynecological position in bed and through palpation, instructions were given to contract the perineal muscles and then repeat this same contraction with the examiner's index and middle fingers introduced into the vagina, considering, according to the Oxford Scale, a variation of 0 to 5 degrees of muscle strength in this palpation. 14 15 These are characterized as 0 - no contraction, 1 - only slight and not sustained contraction, 2 - low intensity contraction with some support, 3 - moderate contraction and there is increased intravaginal pressure (compression of the fingers and some elevation of the vaginal wall), 4 - good contraction (moderate - possible to squeeze the examiner's fingers) and the elevation of the vaginal wall goes towards the symphysis pubis, and 5 - strong, sustained and maintained contraction towards the symphysis pubis. 15

Peripheral muscle strength was measured by dynamometry using palmar grip strength assessed by an upper limb dynamometer (eClear, EH101). Thus, the patients were positioned with the headboard at 90 degrees to the bed, using the dominant upper limb adducted parallel to the trunk, with elbow flexion at 90 degrees and forearm and wrist in neutral position. The pregnant woman was instructed to press the handgrip with her maximum force and no external body movement. The measurement was performed three times, thus using the highest value. 16

And, finally, the functionality evaluation was performed using the Time Up and Go (TUG) test, which consists in measuring, in seconds, the time needed by the patient to get up from a standard armchair, with a height of approximately 46 cm, and walk a distance of 3 m, go around the cone, and return to the chair sitting down again, thus evaluating the functional mobility. 17

All data were stored and analyzed in the software Statistical Package for the Social Sciences for Windows (SPSS) 20.0, and treated with descriptive analysis through mean and standard deviation and categorical data with absolute and percentage values. Intra-group data were evaluated using Student's t-test for repeated measures, and, inter-group comparison using Student's t-test for independent samples. Spearman's Correlation test was used for the correlation of the variables. The significance level adopted was 5%.

## Results

The sample was composed of 72 pregnant women, 36 in the normal parturition group and 36 in the cesarean group. The characterization of the sample is described in Table 1 . The mean age of the women in the normal group was 25.56 ± 6.28 versus 28.57 ± 6.47 for the cesarean section. When comparing the ratio of pre-delivery weight and parturition performed, women with higher weight 86.35 ± 14.86 kg performed cesarean section versus 73.38 ± 11.84 kg in normal parturition (p <0.001). It was also noted that babies born with a lower birth weight of 3.08 ± 0.49kg were conceived by normal parturition and those with a higher birth weight of 3.43 ± 0.50kg by cesarean section (p <0.001).

**Table 1:** Characterization of the sample

Variables	Puerperae Normal Parturition (n = 36)	Puerperae Cesarean Births (n = 36)	p-value
Age (years)	25,56 ± 6,28	28,57 ± 6,47	0,051
Height (m)	1,6 ± 0,05	1,62 ± 0,07	0,167
Prepartum weight (Kg)	73,38 ± 11,84	86,35 ± 14,86	0,001
Pregestational weight (Kg)	62,59 ± 12,08	72,69 ± 14,21	0,017
Newborn length (cm)	48,21 ± 2,29	48,80 ± 1,91	0,217
Newborn weight (Kg)	3,08 ± 0,49	3,43 ± 0,50	0,001
Gestational age (weeks)	39,00 ± 1,18	39,00 ± 1,19	1.00

Values described as mean ± standard deviation; m= meters; Kg= kilograms; RN= newborn; cm= centimeters.

When muscle strength and functionality were compared ( Table 2 ), postpartum women who had vaginal parturition showed a decrease in pelvic floor strength of 1.36 ± 1.11 compared to a cesarean of 2.17 ± 1.17 (p <0.002). On the other hand, women who had a vaginal delivery were more functional when compared to the TUG of 13.21 ± 4.85s compared to those who had a cesarean delivery of 21.17 ± 10.85s (p <0.001). As for the respiratory muscle strength and peripheral strength tests, there was no significant difference in relation to the type of parturition.

**Table 2:** Comparison of muscle strength and functionality

Variable	Puerperae Normal Parturition (n = 36)	Puerperae Cesarean Births (n = 36)	p-value
PFF	1,36 ± 1,11	2,17 ± 1,17	0,002
MEP (cmH20)	33,42 ± 18,40	25,46 ± 10,76	0,027
MIP (cmH20)	33,44 ± 16,29	33,17 ± 13,29	0,913
Handgrip (Kgf)	22,78 ± 5,71	24,31 ± 5,27	0,217
TUG (s)	13,21 ± 4,85	21,17 ± 10,85	0,001

Values described as mean ± standard deviation; with a significance level of p > 0.05; m= meters; PFF = pelvic floor functionality assessment; MEP= maximal expiratory pressure; MIP = maximal inspiratory pressure; cmH2O= centimeters of water; Kgf = kilograms of force; TUG= Time Up Go; s= seconds.

## Discussion

The present study found that women who had a normal parturition are more functional compared to those with a cesarean parturition, but FPA is more decreased after normal delivery than in cesarean parturition. And yet, we demonstrated that the muscle strength and functionality tests performed on women in the immediate postpartum period were safe and feasible since there was no need for any interruption, nor the presence of adverse events associated with the execution of the evaluations.

In our study sample, most of the women who had higher prepartum BMI had a cesarean section, not a normal parturition. Trajner-Bregar et al. conducted a study whose objective was to verify whether the maternal pre-pregnancy weight and weight gain during pregnancy were associated with increased cesarean rates, and their results are similar to ours, concluding that the combination of pre-pregnancy BMI and weight at the time before giving birth is an important determinant of cesarean rates among women. 18 Another study that corroborates our findings is by Li et al. 19 who evaluated the associations between pregestational BMI and weight gain in pregnant women in China alone, and their data revealed that high maternal pregestational BMI and excessive weight gain during pregnancy were associated with cesarean parturition.

Another significant finding of our study was in relation to the weight of the newborn, showing that women in our sample who gave birth to lower birth weight babies, in most cases, had a normal parturition and respectively, of higher weight by cesarean. In a literature review conducted by the Brazilian Medical Association, with the objective of updating the indication for cesarean section and verifying neonatal and perinatal morbidity and mortality in relation to small-for-gestational-age pregnancies, it showed that there is insufficient evidence to recommend planned cesarean section in small-for-gestational-age pregnancies. 20

Regarding the comparison of PFF and type of parturition, we demonstrated in this study that women who underwent cesarean had a higher PFF compared to those who chose natural. Barbosa et al., 4 in their study, analyzed the influence of the route of parturition on pelvic floor strength; the test used in their study was the same used in ours, and their data agree with our results when they conclude that in their sample vaginal partum decreased PFF in primiparous women when compared to cases that underwent cesarean section and to nulliparous women. 4

In a recent systematic review, the impact of the type of parturition on pelvic floor muscles was evaluated by 3D ultrasound, corroborating our data, vaginal parturition showed a decrease in muscle strength in this region, besides being associated with changes in the levator ani muscle, bladder neck mobility, and increased hiatal area, thus presenting a risk factor for prolapse and urinary incontinence. 21 Riesco et al. 22 found that there is a positive correlation between the evaluation of pelvic floor strength when checked with perineometry and/or with digital vaginal palpation, emphasizing the efficacy of the method chosen by us to evaluate PFF; in the same study, they also point out that in pregnancy, changes in PFF generally occurs due to the overload during the gestational period. 22

Barbosa et al. 4 pointed out that regardless of the choice of parturition route, the increase in maternal body weight and the weight of the pregnant uterus will increase the pressure on the pelvic floor muscles during pregnancy, overloading them. A study that evaluated pelvic floor function in 90 pregnant women using perineometry and PFF, with a mean age of 27.6 years, showed that half of the evaluated pregnant women over 30 years of age had grade 5 in PFF and that overweight pregnant women were five times more likely to achieve only grade 3 when compared to pregnant women with BMI within normal values. 15

The mode of birth is linked to a woman's recovery time and therefore may contribute to functional limitations. 23 Vaginal parturition can result in trauma and perineal discomfort and the incision area of a cesarean section is a predisposing factor to morbidity in the puerperium. 24 Some studies suggest that the TUG is a functional test that is simple and can be applied with few resources and is able to assess and quantify functional performance, with basic mobility skills included. 25 26 27 In a study that evaluated 106 primiparous puerperae, 53 parturition vaginally and 53 by cesarean section, it was shown that puerperae who had a vaginal delivery needed less time to perform the TUG, demonstrating a better functional performance in a vaginal parturition, corroborating our findings where women who had a cesarean section were less functional than women who had a vaginal parturition. 25 However, it is noteworthy that puerperal women present a worse result in the TUG test regardless of the type of delivery, when compared to non-parturient women, revealing that functionality in these puerperae is affected, and this result may interfere with their ADLs after leaving the hospital, associating them with mortality, quality of life (QoL) and also with risk of falls. 27

There is still a great need for research in this area, but our findings may help in determining functional disability, identifying potential patients who would need physical therapy treatments, and helping to determine the appropriate treatment for each type of patient. This study has the positive point of being one of the only ones to evaluate respiratory, peripheral, and pelvic floor muscle strength, as well as functionality in puerperal women. Knowledge of the changes in these variables in this population is extremely important in order to reduce complications during hospitalization and ensure functional discharge of patients. A possible negative point was the lack of studies present in the literature for direct comparison with the study population, thus requiring comparison with another population.

## Conclusion

Based on the findings, it was found that there is a reduction in pelvic floor strength in the immediate postpartum period of vaginal parturition in relation to cesarean, on the other hand, women who underwent normal parturition are more functional in relation to those who underwent natural. Respiratory and peripheral muscle strength did not change regardless of the type of parturition.

## References

[1] Leite A C Araújo K K Diástase dos retos abdominais em puérperas e sua relação com variáveis obstétricas Fisioter Mov 2012 25 02 389 397 10.1590/S0103-51502012000200017

[2] Narciso F V Resende A P Bernardes B T Avaliação da função dos músculos do assoalho pélvico de puérperas Fisioter Bras 2010 11 05 324 329 10.33233/fb.v11i5.1416

[3] Michelowski A C Simão L R Melo E C A eficácia da cinesioterapia na redução da diástase do músculo reto abdominal em puérperas de um hospital público em Feira de Santana – BA Rev Bras Saúde Funcional. 2014 2 02 5 16

[4] Barbosa A M Carvalho L R Martins A M [The influence of the delivery route on pelvic floor muscle strength] Rev Bras Ginecol Obstet 2005 27 11 677 682 10.1590/S0100-72032005001100008Portuguese.

[5] Lemos A Caminha M A Melo E F Jr Domelas de Andrade A Avaliação da força muscular respiratória no terceiro trimestre de gestação Rev Bras Fisioter. 2005 9 02 151 156 10.33233/fb.v9i3.1641

[6] Minetto A I Tiago W S Biella M S Victor E G Avaliação da função respiratória em gestantes no projeto interdisciplinar PAMIF (Programa de Atenção Materno-Infantil e Familiar) entre o segundo e terceiro trimestre gestacional Rev Inova Saúde. 2013 2 02 1 15

[7] Costa K NAnálise comparativa da força muscular respiratório em puérperas submetidas a partos transvaginal e transabdomina [trabalho de conclusão do curso]. Campina Grande: Universidade Estadual da Paraíba;2012

[8] Pinto A V Schleder J C Penteado C Gallo R B Avaliação da mecânica respiratória em gestantes Fisioter Pesqui 2015 22 04 348 354. Doi: 10.590/1809-2950/13667922042015

[9] Pereira T RC Souza F G Beleza A CS Implications of pain in functional activities in immediate postpartum period according to the mode of delivery and parity: an observational study Braz J Phys Ther 2017 21 01 37 43 10.1016/j.bjpt.2016.12.003 28442073 PMC5537436

[10] Francisco A A Kinjo M H Bosco C S Silva R L Mendes E P Oliveira S M Associação entre trauma perineal e dor em primíparas Rev Esc Enferm USP 2014 48(Spe):40 45 10.1590/S0080-623420140000600006 25517833

[11] Riesco M L Costa A S Almeida S Basile A L Oliveira S M Episiotomia, laceração e integridade perineal em partos normais: análise de fatores associado Rev Enferm UERJ 2011 19 01 77 83

[12] Sell S E Beresford P C Dias H H Garcia O R Santos E K Olhares e saberes: vivências de puérperas e equipe de enfermagem frente à dor pós-cesariana Texto Contexto Enferm 2012 21 04 766 774 10.1590/S0104-07072012000400006

[13] Bezerra M A Nunes P C Lemos A Força muscular respiratória: comparação entre nuligestas e primigestas Fisioter Pesqui 2011 18 03 235 240 10.1590/S1809-29502011000300006

[14] Paiva D N Bordin D F Gass R Avaliação da força de preensão palmar e dos volumes pulmonares de pacientes hospitalizados por condições não cirúrgicas Sci Med 2014 24 01 61 67 10.15448/1980-6108.2014.1.15402

[15] Correggio K S Trapani Júnior A Correggio K S Mantovani P R Avaliação da função muscular perineal em gestantes Arq Catarin Med 2010 39 03 29 33

[16] Dias J A Ovando A C Kulkamp W Borges N G Junior Força de preensão palmar: métodos de avaliação e fatores que influenciam a medida Rev Bras Cineantropom Desempenho Hum 2010 12 03 209 216 10.5007/1980-0037.2010v12n3p209

[17] Nicolini-Panisson R D Donadio M V Teste Timed “Up & Go” em crianças e adolescentes Rev Paul Pediatr 2013 31 03 377 383 10.1590/S0103-05822013000300016 24142322 PMC4182976

[18] Trojner-Bregar A Blickstein I Lucovnik M Steblovnik L Verdenik I Tul N The relationship between cesarean section rate in term singleton pregnancies, maternal weight, and weight gain during pregnancy J Perinat Med 2016 44 04 393 396 10.1515/jpm-2015-0117 26352070

[19] Li N Liu E Guo J Maternal prepregnancy body mass index and gestational weight gain on pregnancy outcomes PLoS One 2013 8 12 e82310 10.1371/journal.pone.0082310 24376527 PMC3869661

[20] Federação Brasileira das Associações de Ginecologia e Obstetrícia Simões R Bernardo W M Salomão A J Baracat E C Cesarean delivery and small newborn for gestational age Rev Assoc Med Bras 2016 62 01 16 20, quiz 14–15 10.1590/1806-9282.62.01.16 27008485

[21] de Araujo C C Coelho S A Stahlschmidt P Juliato C RT Does vaginal delivery cause more damage to the pelvic floor than cesarean section as determined by 3D ultrasound evaluation? A systematic review Int Urogynecol J Pelvic Floor Dysfunct 2018 29 05 639 645 10.1007/s00192-018-3609-3 29564512

[22] Riesco M L Caroci A S Oliveira S M Lopes M H Avaliação da força muscular perineal durante a gestação e pós parto correlação entre perineometria e palpação digital vaginal Rev Latino-Am Enfermagem 2010 18 06 1138 1144 10.1590/S0104-11692010000600014 21340279

[23] Francisco A A de Oliveira S M Leventhal L C de Bosco C S Crioterapia no pós-parto: tempo de aplicação e mudanças na temperatura perineal Rev Esc Enferm USP 2013 47 03 555 561 10.1590/S0080-623420130000300005 24601129

[24] Mannion C A Vinturache A E McDonald S W Tough S C The influence of back pain and urinary incontinence on daily tasks of mothers at 12 months postpartum PLoS One 2015 10 06 e0129615 10.1371/journal.pone.0129615 26083252 PMC4471341

[25] Santos P L Rett M T Lotti R C Moccellin A S DeSantana J M Via de parto interfere nas atividades cotidianas no puerpério imediato ConScientiae Saúde. 2016 15 04 604 611 10.5585/conssaude.v15n4.6672

[26] Alexandre T S Meira D M Rico N C Mizuta S K Accuracy of Timed Up and Go Test for screening risk of falls among community-dwelling elderly. Rev Bras Fisioter 2012 16 05 381 8 10.1590/s1413-35552012005000041 22858735

[27] Lopes M L Santos J P Fernandes K B Rogério F R Freitas R Q Pires-Oliveira D A Relação da pressão plantar e amplitude de movimento de membros inferiores com o risco de quedas em idosas Fisioter Pesqui 2016 23 02 172 177 10.1590/1809-2950/14871123022016

